# Unravelling the spatial variation of nitrous oxide emissions from a step-feed plug-flow full scale wastewater treatment plant

**DOI:** 10.1038/srep20792

**Published:** 2016-02-08

**Authors:** Yuting Pan, Ben van den Akker, Liu Ye, Bing-Jie Ni, Shane Watts, Katherine Reid, Zhiguo Yuan

**Affiliations:** 1 Advanced Wastewater Management Centre, The University of Queensland, St. Lucia, QLD, Australia; 2 Australian Water Quality Centre, Adelaide, 5000, South Australia, Australia; 3 School of Chemical Engineering, The University of Queensland, St. Lucia, Brisbane, QLD, 4072, Australia; 4 Department of Environmental Science and Engineering, School of Architecture and Environment, Sichuan University, Chengdu, Sichuan 610065, China; 5 Health and Environment Group, School of the Environment, Flinders University, Bedford Park, 5042, South Australia, Australia; 6 Centre for Water Management and Reuse, School of Natural and Built Environments, University of South Australia, Mawson Lakes, 5095, South Australia, Australia

## Abstract

Plug-flow activated sludge reactors (ASR) that are step-feed with wastewater are widely adopted in wastewater treatment plants (WWTPs) due to their ability to maximise the use of the organic carbon in wastewater for denitrification. Nitrous oxide (N_2_O) emissions are expected to vary along these reactors due to pronounced spatial variations in both biomass and substrate concentrations. However, to date, no detailed studies have characterised the impact of the step-feed configuration on emission variability. Here we report on the results from a comprehensive online N_2_O monitoring campaign, which used multiple gas collection hoods to simultaneously measure emission along the length of a full-scale, step-fed, plug-flow ASR in Australia. The measured N_2_O fluxes exhibited strong spatial-temporal variation along the reactor path. The step-feed configuration had a substantial influence on the N_2_O emissions, where the N_2_O emission factors in sections following the first and second step feed were 0.68% ± 0.09% and 3.5% ± 0.49% of the nitrogen load applied to each section. The relatively high biomass-specific nitrogen loading rate in the second section of the reactor was most likely cause of the high emissions from this section.

Nitrous oxide (N_2_O) is a potent greenhouse gas (GHG), with an approximately 300-fold stronger global warming effect than carbon dioxide^1^. In wastewater treatment plants, N_2_O is mainly produced and emitted during biological nitrogen removal (BNR) process. The overall carbon footprint of a WWTP is highly sensitive to N_2_O emission. For example, De Haas and Hartley^2^ estimated that the carbon footprint of a WWTP would increase by approximately 30% if N_2_O emission represented 1% of the nitrogen denitrified (or approximately 0.5% of the nitrogen load). Therefore, understanding N_2_O emissions is of great importance to the operation of WWTPs, particularly as regulations are introduced to develop emission inventories and control strategies to reduce net environmental impacts.

In the past few years, there have been significant efforts worldwide to quantify and investigate N_2_O emissions from full-scale BNR processes. The methods used have evolved from the initial grab-sampling based method^3^ to continuous online monitoring method, which has been now widely-adopted^4^,^5^. The latter is typically done by monitoring the N_2_O concentration and flow rate of gases over the operational range of the BNR process using portable online instruments. Many WWTPs are not enclosed and therefore floating hoods are often used to cover a small portion (e.g. 0.13 m^2^–0.6 m^2^) of the reactors surface to capture a representative gas sample^4^,^6^,^7^. This approach is able to capture the diurnal and long-term temporal dynamics in the N_2_O emission fluxes, which provides a more reliable means to quantify N_2_O emissions.

Continuous online monitoring has revealed that N_2_O emissions from wastewater treatment systems are highly dynamic^8^,^9^. Variation in several factors are believed to influence emission dynamics, which include the nitrogen loading rate^4^, dissolved oxygen (DO)^5^ and nitrite concentrations^6^. It has further been reported that N_2_O is primarily emitted from the aerated zones of a reactor^10^. This is attributed to the intensive stripping of N_2_O as it is being produced^10^. In comparison, negligible emissions have been observed from non-aerated zones due to the lack of active stripping^4^. N_2_O production during wastewater treatment is primarily biological, with nitrifying and denitrifying microorganisms the primary facilitators within the aerobic zone and anoxic zones respectively, with the dominant source of N_2_O believed to be generated by ammonia-oxidising microorganisms^4^,^9^.

In addition to temporal fluctuations in N_2_O emissions, strong spatial variation in emission has been previously reported; particularly for large plug-flow reactors given steep spatial gradients in concentrations of DO and nitrogen species can exist along the reactors path^4^,^5^,^7^,^11^. These spatial variations in nitrogen and oxygen concentrations are highest plug flow reactors that are step-fed, whereby organic rich wastewater is re-introduced at a second stage to drive denitrification^12^. However, in addition to spatial gradients in substrate concentrations the step-feed strategy also produces a gradient of biomass along the length of the reactor given the returned activated sludge (RAS) stream is typically fed only to the beginning of the reactor. As a result the biomass concentration is higher in the upstream sections than compared with the downstream sections because the biomass becomes diluted by the second step-feed. This results in an uneven biomass-specific nitrogen loading along the reactor. It is possible that this gradient in biomass concentration would cause further spatial variations in the N_2_O fluxes, given previous studies have shown that the biomass-specific nitrogen loading rate has a strong influence on N_2_O production^4^,^13^. However to date, the effect of such a feeding strategy on N_2_O emission has not been reported.

The need to characterise spatial variability in emissions is required not only to better quantify the plants emission factor, but to understand the key drivers for N_2_O production and identify control measures. This would require an online monitoring approach that can measure N_2_O concentrations at multiple locations. Accordingly, the overall aim of this study was twofold: 1) to characterise the spatial variation of N_2_O emissions from a full-scale plug-flow WWTP by developing a novel online method that can sequentially measure N_2_O concentrations in the off-gas from multiple locations; and 2) to investigate the effect that a step-feed configuration ASR has on N_2_O emissions. To this end, a comprehensive online monitoring program was undertaken to quantify N_2_O emissions at multiple sampling locations positioned along the aerobic zones of a plug-flow step-fed reactor. To complement the online monitoring program, an intensive manual sampling campaign was conducted whereby hourly mixed liquor grab samples were taken from multiple locations across the reactors over a four day period (daytime only), for liquid-phase analysis of N_2_O and other inorganic nitrogen species. Further, the ammonium and Total Kjeldahl Nitrogen (TKN) concentrations in the influent were also measured to calculate the N_2_O emission factors for the different steps.

## Results

### Wastewater characteristics and plant performance

The characteristics of the ASRs influent and effluent during the 7-week monitoring period are shown in Table 1. The average TCOD and TKN removal efficiencies were around 90% and 75%, respectively. Non-biodegradable COD was the main form of organic matter in the effluent, with an average concentration of 48 mg/L. Nitrate was the main nitrogen product in the effluent, with an average concentration of 12 mg N/L. The effluent N_2_O concentration was typically below 0.1 mg N/L, and was one order of magnitude higher than concentrations detected within the influent (Table 1).

### Diurnal and spatial variation of N_2_O emissions

**Figure 1:**
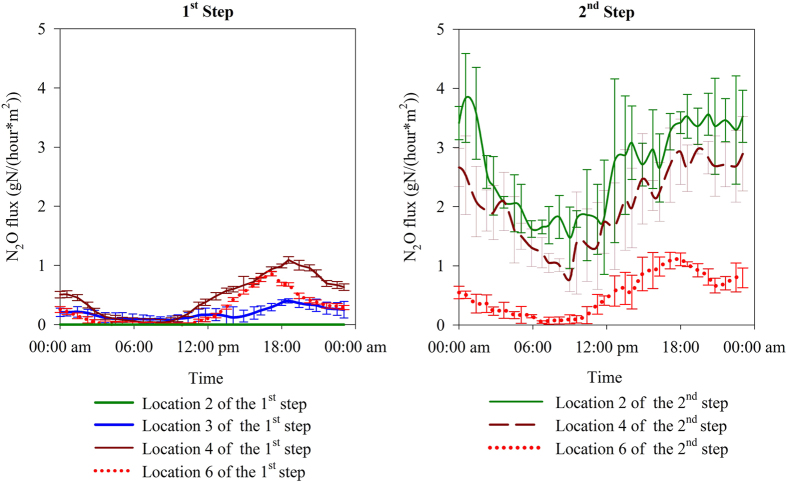
The average diurnal N_2_O flux profiles from the 1^st^ and the 2^nd^ Step (error bars showing standard deviations).

The long-term online monitoring showed that the N_2_O emissions were highly dynamic; however, a reoccurring diurnal pattern of N_2_O emission profiles was evident across all locations. Figure 1 shows the diurnal N_2_O emission profiles at all six monitored locations across the two steps. The profiles generally followed a pattern with an “N_2_O emission valley” in the morning and an “N_2_O emission peak” after 18:00 pm. This pattern roughly mirrored the diurnal pattern of the influent flow rate as shown in Figure S1 within the Supplementary Content.

A high level of spatial variability of N_2_O fluxes was also clearly observed along the reactor. For the 1^st^ step, N_2_O emission was negligible at Location 2 (the very beginning of the aeration zone). This is contradictory to some previous observations that N_2_O emission tended to increase within this transition zone^6^,^14^. Considerable amount of N_2_O emission was observed at Location 3 (25 meters from the beginning of the aeration zone), with the peak flux measured at 0.5 g N/(hour × m^2^). The highest N_2_O emission was observed at Location 4 (50 meters from the beginning of the aeration zone) with the peak flux measured at 1.2 g N/(hour × m^2^). High N_2_O emissions were also observed at the end of aeration zone of the 1^st^ step (Location 6, which was 155 meters away from the beginning of the aeration zone), where the peak flux was 1.1 g N/(hour × m^2^). However, the N_2_O emission peaks from the middle of the aeration zone (Location 4) were much higher and wider than peaks from the end of the aeration zone (Location 6).

The spatial variation of N_2_O fluxes from the 2^nd^ step showed a different pattern in comparison to that of the 1^st^ step. The highest N_2_O emission was observed immediately after the anoxic zone (Location 2) with peak flux values measured around 4 g N/(hour × m^2^). The N_2_O emission flux reduced slightly at Location 4 (25 meters from the beginning of the aeration zone), and had reduced considerably at Location 6 (80 meters from the beginning of the aeration zone).

The N_2_O fluxes measured within the 2^nd^ step were significantly higher than the fluxes measured at the equivalent locations in the 1^st^ step, particularly from the beginning and middle sections. At Locations 2 and 4 of the 2^nd^ step, the N_2_O fluxes were well above 1 g N/(hour × m^2^) and typically reached 3.5 g N/(hour × m^2^) for the majority of a day (>14 hours). In comparison, the N_2_O emission fluxes at the equivalent locations along the 1^st^ step were consistently lower than 1 g N/(hour × m^2^).

### Spatial variation of dissolved N_2_O

**Figure 2:**
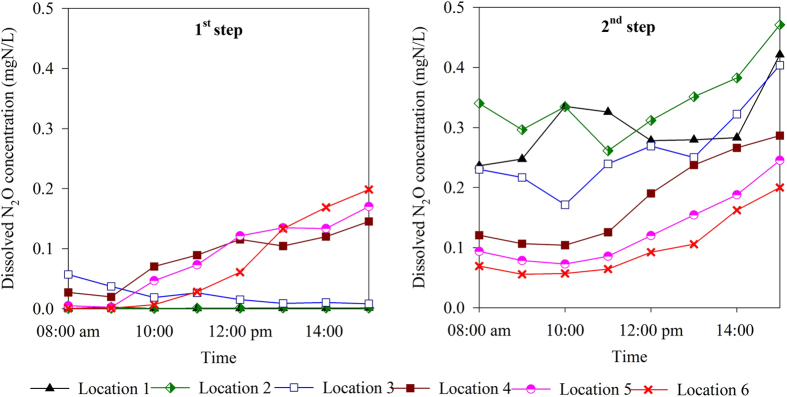
Liquid phase N_2_O concentration in the 1^st^ step and in the 2^nd^ step.

Dissolved N_2_O concentration in the influent was determined to be 0.0012 ± 0.00075 mg N/L, which is within the previously reported ranges^15^. The dissolved N_2_O concentration measured within the reactor (Fig. 2) was much higher than the N_2_O concentration in the influent, which confirms that a significant amount of N_2_O was produced during the BNR process. For each location, except for Location 2 of the 1^st^ step, the dissolved N_2_O concentration gradually increased from 8:00 am (when the manual sampling started) to 15:00 pm (when the manual sampling stopped). This ascending trend was in line with the gaseous N_2_O emission trend shown in Fig. 1.

Similar to the online gaseous N_2_O monitoring data, the spatial variability of dissolved N_2_O concentration was substantial and the two steps display different patterns.

In the 1^st^ step, negligible N_2_O was found in the anoxic zone (Location 1) and at the beginning of the aeration zone (Location 2). This observation is consistent with the fact that no gaseous N_2_O emission was observed at Location 2 of the 1^st^ step (Fig. 1). The N_2_O concentration gradually increased along the path of the plug-flow reactor of the 1^st^ step. The highest N_2_O concentrations were observed downstream at Locations 4, 5 and 6, where values ranged between 0.1 to 0.2 mg N/L at 3 pm.

Conversely, in the 2^nd^ step, the dissolved N_2_O concentration gradually reduced along the path of the plug-flow reactor. The highest N_2_O concentration was observed in the anoxic zone (Location 1) and at the beginning of aeration zone (Location 2 and Location 3). The peak values at these locations were also observed at 3 pm, where values reached 0.4 to 0.5 mg N/L. The lowest dissolved N_2_O concentration was observed at Location 6, which ranged between 0.06 and 0.19 mg N/L.

Overall, the dissolved N_2_O concentration in the 2^nd^ step was much higher than the 1^st^ step, which was in line with the online gaseous N_2_O emission profile.

### Spatial variation of other parameters relevant to N_2_O emissions

**Figure 3:**
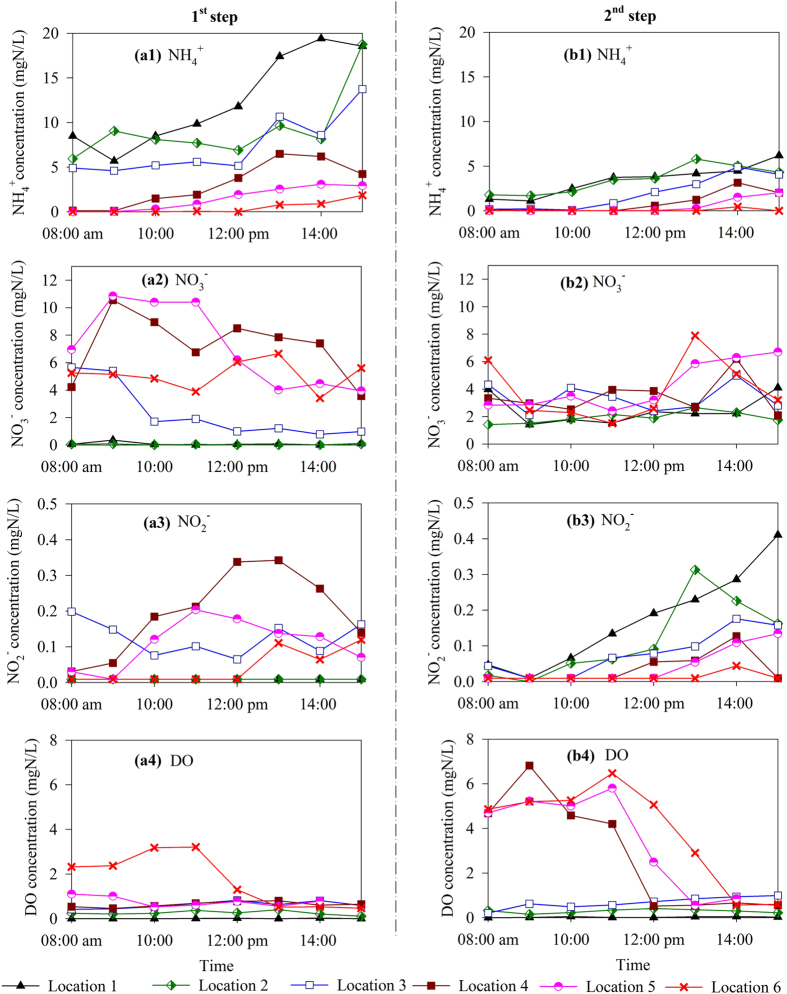
Liquid phase concentration profiles of NH_4_^+^, NO_3_^−^, NO_2_^−^ and DO in the 1^st^ step and in the 2^nd^ step.

Several factors, such as DO level^16^,^17^,^18^, nitrite or free nitrous acid (FNA) concentration^19^,^20^, pH level^21^,^22^ have been shown to affect N_2_O production. Therefore, the spatial variations of these parameters, together with the NH_4_^+^and NO_3_^−^ concentrations, were monitored. The results are summarized in Fig. 3.

The spatial variation of NH_4_^+^ and NO_3_^−^ concentrations seen here were expected for this type of reactor configuration. The NH_4_^+^ concentration gradually reduced from Location 2 to Location 6 in both steps (Fig. 3 a1 & b1), which was coupled with a gradual increase in the NO_3_^−^ concentration due to nitrification (Fig. 3 a2 & b2).

The nitrite levels in the 1^st^ step and in the 2^nd^ step showed very different trends (Fig. 3 a3 & b3). In the 1^st^ step, negligible NO_2_^−^ was found in the anoxic zone (Location 1) and at the beginning of the aeration zone (Location 2). Higher NO_2_^−^ concentrations (up to 0.35 mg N/L) were observed at downstream locations in the reactor (Locations 3, 4, 5 and 6). In contrast, the NO_2_^−^ concentrations along the 2^nd^ step gradually reduced along the path of the plug-flow reactor, with the highest NO_2_^−^ concentration (up to 0.41 mg N/L) observed within the anoxic zone (Location 1) and at the beginning of aeration zone (Location 2).

The DO concentrations in the 1^st^ and the 2^nd^ steps showed very similar trends (Fig. 3 a4 & b4). Negligible DO concentration was detected in the anoxic zones (Location 1) of both steps. The DO concentration increased along the length of the aerobic zone (from locations 2 to 6) as COD and nitrogen substrates were oxidised and the aeration flow rate was kept steady. This ascending trend of DO along the reactor was more obvious in the morning (Location 6 of the 1^st^ step and Locations 4, 5, 6 of the 2^nd^ step), because the ammonium substrate was readily exhausted during this period of low loading (Fig. 3 b1).

## Discussion

### N_2_O emission factor from the step-feed plug-flow system

To date, monitoring of many full-scale plants has been carried out with the awareness of N_2_O as a potent greenhouse gas. Early investigations relied on the grab sampling method, which yielded highly variable N_2_O emission factors with values ranging between 0.6% to 25% of the influent nitrogen load^3^. Due to the recent adoption of more reliable online monitoring methods, the N_2_O emission factor has been refined and narrowed down greatly, with the reported emission factors for full-scale BNR plants varying between 0.01% to 6.8%^4^,^5^,^7^,^11^,^23^,^24^,^25^. In this study, the combined N_2_O emission factor of the two-step feed, plug-flow system was determined to be 1.9% ± 0.25%, which is among the highest values reported so far. For comparison, a literature review of reported N_2_O emissions from full-scale wastewater treatment plants is shown Table S1 in Supplementary Content.

As shown in Fig. 1, significant temporal and spatial variability in the N_2_O fluxes were observed along the plug-flow path. The calculated N_2_O emission factor for each step, revealed that the step-feed configuration exerts a substantial influence on the N_2_O emission, given that the N_2_O emission factor from the first and second steps differed substantially, measuring 0.68% ± 0.09% and 3.5% ± 0.49%, respectively, of the influent nitrogen loading to each path (Table 2). This result suggests that it is crucial to consider spatial variations of N_2_O emission when quantifying emission factors from plug-flow systems.

For the majority of open reactors, characterising the spatial variability and emission ‘hotspots’ seen here presents a challenge, which was easily overcome by sequentially measuring N_2_O concentrations in the off-gas from multiple hoods. Capturing this information was crucial to accurately quantify the reactors overall emission factor. Fully enclosed aeration basins would also benefit from this approach given the ability to identify hotspots and relate these with bulk water quality parameters can be used to identify the mechanisms responsible for N_2_O production and hence allow more targeted performance optimisation measures to be made. This information would otherwise be missed if monitoring was focused solely on measuring the concentration of N_2_O in the bulk off-gas from a common collection point.

### Likely mechanism of N_2_O emission variations in the step-feed plug-flow system

The major N_2_O production pathways during BNR process include the hydroxylamine oxidation pathway of ammonia oxidizing bacteria (AOB), nitrifier denitrification pathway of AOB, heterotrophic denitrification pathway and chemical reactions^26^,^27^. The interplay of many parameters (such as DO and nitrite concentrations) determines the functional N_2_O production pathways as well as the overall emission factor^28^,^29^.

For the plant studied, the majority of N_2_O that was emitted from the 1^st^ step was generated in the aerobic zone, which was likely due to the nitrification process. This conclusion is based on the fact that there was negligible N_2_O accumulation in the anoxic zone of the 1^st^ step (Fig. 2) as well as negligible N_2_O emission at the beginning of the aeration zone (Location 2) (Fig. 1). The negligible level of nitrate and nitrite at Location 1 (Fig. 3) showed that the anoxic zone in the 1^st^ step was able to completely remove the small amount of oxidised nitrogen that was introduced by the RAS stream, which resulted in no N_2_O accumulation from heterotrophic denitrification. Therefore, the anoxic zone of the 1^st^ step played a very minor role in the overall N_2_O emission factor.

In contrast to the 1^st^ step, N_2_O was generated in both the anoxic and aerobic zones of the 2^nd^ step. N_2_O accumulated to concentrations as high as 0.5 mg N/L at Location 1 in the anoxic zone of the 2^nd^ step, indicating that there was N_2_O accumulation during denitrification in the anoxic zone. The N_2_O accumulated in the anoxic zone was subsequently stripped at the beginning of the following aerobic zone, which was captured by the hood located at Location 3. As shown in Fig. 3, in contrast to the negligible nitrate and nitrite concentrations in the anoxic zone in the 1^st^ step, the nitrate and nitrite concentrations in the anoxic zone in the 2^nd^ step reached 4.0 and 0.4 mg N/L respectively. This indicated that denitrification in the anoxic zone in the 2nd step was incomplete; a condition favouring N_2_O accumulation^30^. Denitrification was likely limited by the carbon source in the wastewater that was fed to this zone, resulting in N_2_O accumulation.

This study revealed that the step-feed strategy could have a significant impact on N_2_O emissions. The 2^nd^ step had a much higher N_2_O emission factor compared to the 1^st^ step (Table 2) (3.5% vs. 0.68%). N_2_O accumulation in the anoxic zone in the 2^nd^ step clearly contributed to the higher emission factor. Based on the flow rate of the mixed liquor in the 2^nd^ Step of 68.1ML/day (27.3 + 22.7 + 18.1  =  68.1ML/day, see section 2.1 for details), and the average dissolved N_2_O concentration in the second anoxic zone of approximately 0.2 mg N/L (estimated based on the dissolved N_2_O concentrations during daytime shown in Fig. 2 and the N_2_O dynamics shown in Fig. 1), it was estimated that approximately 13.6 kg N/day of N_2_O produced in the anoxic zone in the 2^nd^ Step would flow into the aerobic zone. By assuming that the full amount was stripped out in the aerobic zone, the anoxic zone’s contribution to the overall N_2_O emission in the second Step would be 27% (13.6 kg N/day ÷ 49.8 N/day, 49.8 N/day is the average daily N_2_O emission from the second step, as detailed in Table 2). This represents an upper limit of the anoxic zone contribution as some of the N_2_O washed into the aerobic zone could potentially be reduced to N_2_ by denitrifiers. Our analysis showed that the AOB were likely to be the primary contributors (>73%) to the high N_2_O emission in the 2^nd^ Step, which exceeded the AOB contribution in the 1^st^ Step.

As shown in Table 2, the average daily N_2_O emission from the 2^nd^ Step was 49.8 kg N/day. Even when assuming all of the N_2_O accumulated in the anoxic zone was stripped (13.6 kg N/day) the N_2_O produced in the aerobic zone was 36.2 kg N/day (49.8 kg N/day-13.6 kg N/day). This amount, which is likely to be highly conservative, was much higher than the N_2_O emitted in the 1^st^ Step (12.5 kg N/day). Therefore, the generation of N_2_O by AOB activity in the 2^nd^ Step was much higher than that in the 1^st^ Step. Previous studies have shown that the biomass specific N_2_O production rate increases as the ammonia oxidation rate increases^31^. This was shown to be true for both the hydroxylamine oxidation pathway and the AOB denitrification pathway^32^. These findings suggest that AOB tend to produce more N_2_O at higher ammonia oxidation rates. In this study, the MLVSS concentration in the 2^nd^ step was around 40% lower than that in the first step because of the dilution effect provided by influent from the 2^nd^ step. The biomass specific nitrogen and COD loafing rate to the 1^st^ step were 0.047 kgN/(kgVSS × day) and 0.36 kgCOD/(kgVSS × day), in comparison to 0.065 kgN/(kgVSS × day) and 0.51 kgCOD/(kgVSS × day), respectively to the 2^nd^ step. This led to a higher F/M (food to microorganism) ratio in the 2^nd^ step and hence, a higher biomass specific ammonia oxidation rate, which favoured higher N_2_O production.

One potential mitigation strategy could be to evenly divide the RAS into anoxic zones of both steps (currently RAS is returned only to the 1^st^ step). In doing so, the MLVSS concentration in the 2^nd^ step would increase, resulting in a lower F/M ratio which may reduce N_2_O production. However, for this scenario N_2_O production in the 1^st^ step is expected to increase, however to a lesser extent, leading to a reduction in the overall N_2_O emission from the system.

## Material and Methods

### Process scheme of the full-scale plug-flow step-feed activated sludge reactor

The ASR used for this case study employed a plug-flow step-feed configuration for biological nitrogen and carbon removal from domestic wastewater, with a design capacity of 50 ML/day. The reactor has a working volume of 21,205 m^3^, with a designed hydraulic retention time (HRT) of 12 h and aerobic sludge retention time (SRT) of 8 days (total SRT of 12 days).

**Figure 4:**
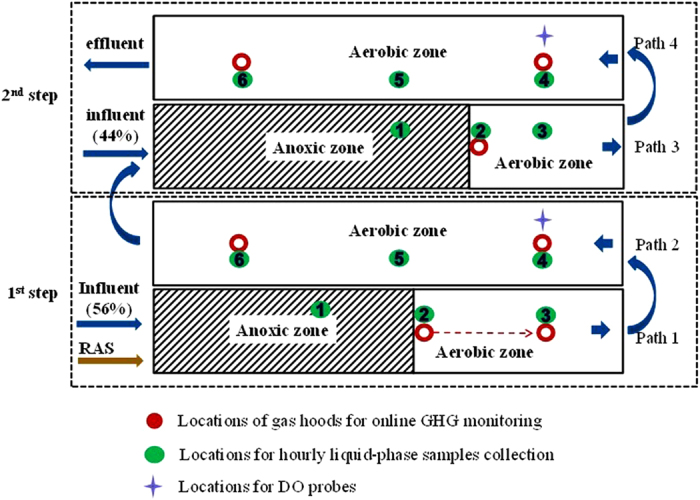
A simplified process flow diagram of the two-step plug-flow BNR reactor studied (the hood originally placed at Location 2 of the 1^st^ step was moved to Location 3 after one-week monitoring, indicated by the arrow).

A simplified process flow diagram of the reactor studied is shown in Fig. 4. The plug-flow reactor consists of four paths, each with a volume of 5340 m^3^ (89m × 12m × 5m). The influent feed is split, where 56% (27.3 ML/day) is fed at the beginning of the 1^st^ step and 44% (22.7 ML/day) is fed in the 2^nd^ step, forming a two-step configuration. The 1^st^ step consists of Path 1 and Path 2 and the 2^nd^ step consists of Path 3 and Path 4. Each step is comprised of an anoxic zone for denitrification (3000 m^3^ for the 1^st^ step and 4440 m^3^ for the 2^nd^ step) followed by an aerobic zone for nitrification. The 2^nd^ step begins with an anoxic zone with a working volume of 4440 m^3^ (74 m×12 m×5 m) for denitrification, followed by a second aerated zone for nitrification. The mixed liquor from Path 2 enters Path 3, which is mixed with the influent fed to this path in the anoxic zone. The effluent exits the reactor at the end of Path 4. The RAS from the secondary clarifiers, with a calculated flow rate of 18.1 ML/day, is recycled to the beginning of the anoxic zone of Path 1. Aeration control is based on online DO measurement with DO probes installed in each step (Fig. 4), with the DO set-point fixed at around 1 mg/L.

### Gas-phase N_2_O monitoring using gas hoods and on-line N_2_O analysers

Multiple sampling locations were chosen in this study to investigate the spatial variation in N_2_O emissions from different paths of the two-step plug-flow BNR reactor. The locations of the gas hoods used to collect the gas-phase N_2_O concentration and gas flow data are as indicated in Fig. 4. These sampling points were specifically chosen to cover the beginning (Locations 2 and 3 of the 1^st^ step, Location 2 of the 2^nd^ step), the middle (Location 4 of each step) and the end (Location 6 of each step) of the aerobic zones. The gas hoods were not placed within the anoxic zones since there was no measureable gas flow here and previous studies have shown that N_2_O fluxes from un-aerated zones are negligible^10^.

The on-line gas-phase N_2_O monitoring was conducted over a seven week period. Three gas hoods were designed and anchored along the aerated zone to allow continuous online emission monitoring. During the first sampling week, the three hoods were placed at Location 2, Location 4 and Location 6 of the 1^st^ step. In the second to the fourth sampling week, the hood originally placed at Location 2 was moved along the reactor to Location 3 of the 1^st^ step, while the other two hoods remained at their same location. Between the fifth to the seventh sampling weeks, the three hoods were moved to the 2^nd^ step, located at Locations 2, 4 and 6.

The three off-gas hoods were modified from plastic commercial hopper tanks. The wall of the hopper tank was shortened to approximately 280 mm, giving a total height of 540 mm (shown in Figure S2 in Supplementary Content). The bottom diameter was 530 mm and covered an area of 0.22 m^2^. The hoods were lowered to allow a minimum depth of 100–150 mm into the water column, resulting in a maximum permissible gas pressure within the hoods of 1.0–1.5 kPa (to keep the wall of hoods submerged). Each of the plastic hopper hoods were attached with a high-density polystyrene skirt to ensure that they floated and were fixed in position using nylon rope secured to three anchor points.

**Figure 5:**
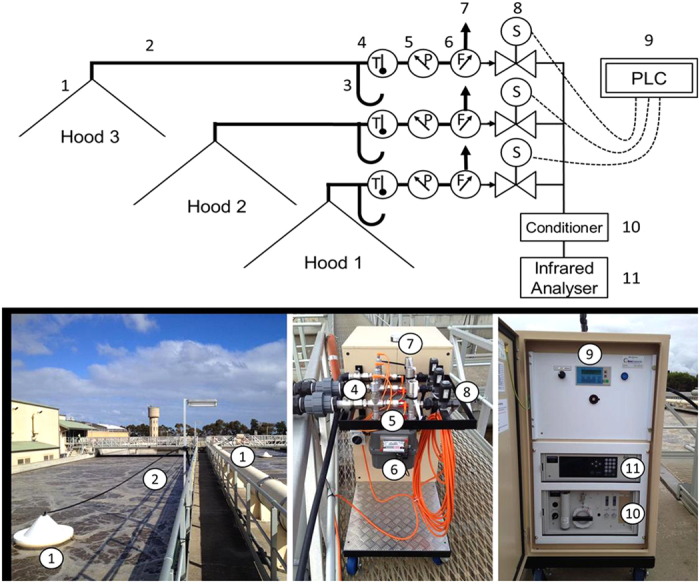
The monitoring system: (**a**) line diagram and (**b**) photograph of the set-up. (**1**) gas-hoods positioned along the aeration zone; (**2**) polyethylene gas tubing; (**3**) condensation moisture trap; (**4**) temperature sensor; (**5**) pressure sensor; (**6**) flow meter; (**7**) excess gas exhaust; (**8**) solenoid gas valve for multiple hood gas sampling; (**9**) programmable logic controller to control solenoid gas valves for gas sampling from each hood; (**10**) gas sample conditioning system; (**11**) infrared N_2_O, CH_4_ and O_2_ gas analyzer.

The off-gas collected from each of the three gas hoods were transferred to a central off-gas monitoring unit, via 50mm diameter polyethylene gas tubing to allow continuous emission monitoring. A detailed description of the off-gas collection and monitoring unit is provided in Fig. 5. Once the off-gas from each of the hoods reached the monitoring unit, gas temperature, pressure and flow rate were measured and recorded in real-time. After the flow meter (Landis + Gyr, mode 750), a small portion of the gas (4 L/min) was diverted and pumped to the gas conditioning unit (Horiba VS3002) and analyzer (Horiba VA3000) via an internal air pump situated within the Horiba analyzer. The excess off-gas (20 – 100 L/min) was continuously exhausted from the outlet of the flow meter. As the analyzer can only measure one gas stream at a time, a Siemens Programmable Logic Controller (PLC) was used to control the cyclic opening and closing of solenoid valves to direct the gas captured from the individual hoods to the analyser at 6 minute intervals. N_2_O concentration (in ppmv) temperature, flow rate and pressure were logged at two minute intervals. The gas analyser had a N_2_O measurement range of 0 to 500 ppmv, with a detection limit of 2 ppmv at an accuracy of 1% of the scale. The analyser was serviced and calibrated on-site, according to manufacturer’s instructions, using compressed air and 450 ppmv N_2_O gas standard (Air Liquide Australia). In addition, other online data recorded by the plant operator, including the influent flow rate, aeration flow rate and DO concentrations were also collected.

### Liquid phase measurements through off-line sampling

The purpose of the grab sampling campaign was to collect liquid-phase N_2_O, as well as NH_4_^+^-N, NO_3_^−^-N, and NO_2_^−^-N data to gain further insight into the N_2_O production at different locations along the reactor. Hourly grab samples (from 8 am to 3 pm) were manually taken from multiple sampling locations shown in Fig. 4, for wastewater and mixed liquor composition analysis (more details in Table 3). These sampling points covered influent, anoxic zone (Location 1) and different locations along the aerobic zones (Location 2 to Location 6). Samples were analyzed for dissolved N_2_O, NH_4_^+^-N, NO_3_^−^-N, and NO_2_^−^-N. The pH, temperature and DO were also measured hourly at these locations using a portable DO/pH/T meter (YSI Professional Plus, United States).

In addition, 24 h composite samples were taken from the influent and effluent using refrigerated automatic samples for the measurement of TCOD, TKN, NH_4_^+^-N, NO_3_^−^-N and NO_2_^−^-N.

### Chemical analysis

The collected liquid samples were immediately filtered with 0.45 mm disposable sterile filters (Millipore, Millex GP) and were subsequently injected into freshly vacuumed Labco Exetainers to allow equilibration of gas and liquid phases. The N_2_O concentrations in the gas phase of the tube were measured using a Shimadzu GC-9A gas chromatograph equipped with a micro-electron capture detector (ECD) and a flame ionization detector (FID), respectively. Each Labco Exetainer tube was weighed before and after sampling to determine the sample volume collected. This volume, along with the known volume of the Exetainers, enables the dissolved N_2_O concentration contained in the original wastewater sample to be calculated^15^. The detection limit of the liquid phase N_2_O concentration is 4.5 × 10^−5 ^mg N/L. The filtered samples were also analysed for the NH_4_^+^, NO_3_^−^ and NO_2_^−^ concentrations using Lachat QuickChem8000 Flow Injection Analyser (Lachat Instrument, Milwaukee, USA). Mixed liquor suspend solid (MLSS) and volatile solids (MLVSS) were measured in triplicates according to the Standard Methods^33^. TCOD and TKN in samples collected were analysed according to Standard Methods^33^.

### Calculation of N_2_O emission

The N_2_O fluxes were calculated based on the online monitoring results of the gas-phase N_2_O concentration, gas flow rate and temperature. The data collected by the three hoods in each step were all considered. The hood located at Location 3 of the 1^st^ step and Location 2 of the 2^nd^ step was used to represent the first 30% surface area of the respective aeration basin. Similarly, the hood located at Location 4 of both steps was used to represent the middle 40% surface area, and the hood located at Location 6 of both steps was used to represent the last 30% surface area. Since there was no measureable gas flow from the anoxic zone, the gas hoods were not placed here. Due to the lack of active stripping, N_2_O emission from non-aerated areas has been found to be negligible in previous studies, and N_2_O accumulated at the anoxic zone has been found to be stripped in the aeration zone in previous studies^10^.

The net N_2_O emitted from each hood covered area over a given a period of time (Δt) were calculated using Eq-1:


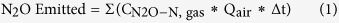


where C_N2O-N, gas_ is the N_2_O-N concentration in the off-gas (mg N_2_O-N/L); Q_air_ is the flow rate of the off-gas (L/hour); Δt is time interval by which the off-gas N_2_O concentration was measured (one minute in this study). The unit of N_2_O concentration in the off-gas was converted from ppmv (directly measured by the on-line analyzer) to mg N_2_O-N/L, and corrected for temperature at the time of sampling.

## Additional Information

**How to cite this article**: Pan, Y. *et al.* Unravelling the spatial variation of nitrous oxide emissions from a step-feed plug-flow full scale wastewater treatment plant. *Sci. Rep.* **6**, 20792; doi: 10.1038/srep20792 (2016).

## Supplementary Material

Supplementary Information

## Figures and Tables

**Table 1:** Influent and effluent characteristics of the two-step plug-flow BNR reactor studied.

	Influent		Effluent	
	Range	Average (±standard deviation)	Range	Average (±standard deviation)
Chemical oxygen demand, COD (mg/L)	345–788	499 ± 104	51–310	54 ± 102
Biological oxygen demand, BOD (mg/L)	88–335	207 ± 54	2–17	6 ± 4
Total Kjeldahl nitrogen, TKN (mg /L)	45.5–78.8	64.0 ± 6.5	2–10	3.3 ± 1.6
Ammonium, NH_4_^+^ (mg N/L)	36.8–54.5	47.4 ± 3.5	0–7	0.3 ± 0.64
Nitrate, NO_3_^−^ (mg N/L)	0–0.85	0.18 ± 0.13	5–19	12.1 ± 5
Nitrite, NO_2_^−^ (mg N/L)	not detectable		0.009–0.19	0.05 ± 0.05
Nitrous oxide, N_2_O (mg N/L)	0–0.0026	0.0012 ± 0.00075	0.0007–0.1984	0.045 ± 0.054

**Table 2:** N_2_O emissions determined for the two-step feed, plug-flow reactor.

	Average daily N_2_O emitted (kg N/day)	Emission factor
Overall plant	62.3	1.9% ± 0.25%
1^st^ step	12.5	0.68% ± 0.09%
2^nd^ step	49.8	3.5% ± 0.49%

**Table 3:** On-line monitoring and offline sampling program.

Monitoring period	Hood location	Liquid phase sampling locations	Liquid phase sampling day
Week 1	the 1^st^ step: Locations 2, 4 & 6	None	None
Week 2 – Week 4	the 1^st^ step: Locations 3, 4 & 6	the 1^st^ step: influent, Locations 1, 2, 3, 4, 5 & 6	Week 4: on Tuesday and Wednesday
Week 5 – Week 7	the 2^nd^ step: Locations 3, 4 & 6	the 2^nd^ step: influent, Locations 1, 2, 3, 4, 5 & 6	Week 5: on Tuesday and Thursday
