# Supplementary material for: Unravelling the spatial variation of nitrous oxide emissions from a step-feed plug-flow full scale wastewater treatment plant
Source: Sci Rep. 2016 Feb 8;6:20792. doi: 10.1038/srep20792 (PMC4745105; doi:10.1038/srep20792)
Supplement: Supplementary Information [file srep20792-s1.pdf]

Appendix Supplementary Material

Unravelling the spatial variation of nitrous oxide emissions from a step-feed plug-flow full scale wastewater treatment plant

Yuting Pan[1,4], Ben van den Akker[2,5,6], Liu Ye[1,3], Bing-Jie Ni[1], Shane Watts[1], Katherine Reid [2], Zhiguo Yuan[1,*]

[1]Advanced Wastewater Management Centre, The University of Queensland, St. Lucia, QLD, Australia

[2]Australian Water Quality Centre, Adelaide, 5000, South Australia

[3]School of Chemical Engineering, The University of Queensland, St. Lucia, Brisbane, QLD 4072, Australia

[4]Department of Environmental Science and Engineering, School of Architecture and Environment, Sichuan University, Chengdu, Sichuan 610065, China

[5]Health and Environment Group, School of the Environment, Flinders University, Bedford Park, 5042, South Australia, Australia.

[6]Centre for Water Management and Reuse, School of Natural and Built Environments, University of South Australia, Mawson Lakes, 5095, South Australia, Australia

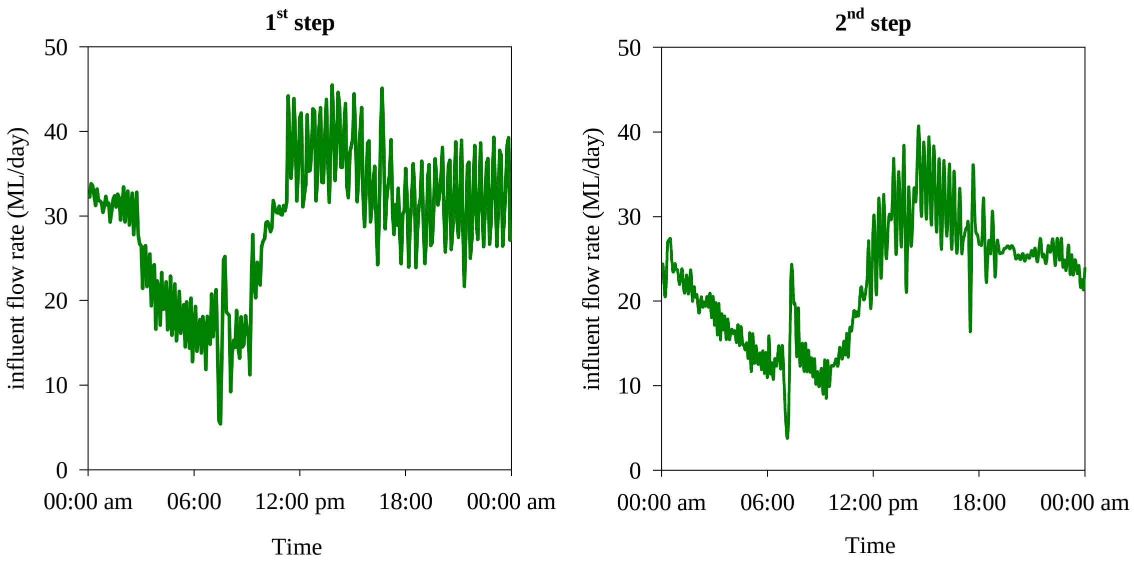

Figure S1: The typical daily influent flow rate profile

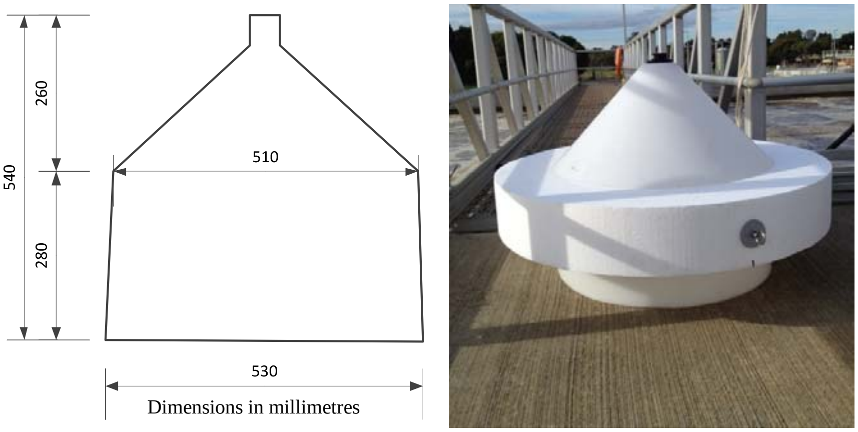

Figure S2: Design of the off-gas collection hood

Table S1. A review of literature reports on N_2O emissions from full-scale conventional wastewater treatment plants

Type of WWTP	N_2O emission factor (% of N-influent) and other key findings	Method to quantify N_2O emission	Reference
**Studies with emission factor estimated based on continuous online monitoring**			
12 biological nitrogen removal (BNR) plants, including the following configurations: two-stage (nitrification-denitrification) BNR, four-stage Bardenpho, step-feed BNR, step-feed non-BNR, Modified Ludzack Ettinger (MLE) process, oxidation ditch	➢ 0.01~1.8%<br>• A high degree of diurnal variability in N_2O emission, correlated with diurnal total Kjeldahl nitrogen loading;<br>• Aerobic zones contributed more to N_2O fluxes than anoxic zones;<br>• In aerobic zones, N_2O emissions were positively correlated to high nitrite, ammonium, and dissolved oxygen concentration;<br>• In anoxic zones, N_2O emissions were positively correlated to high nitrite and oxygen concentration.	o Continuous, online monitoring of gaseous N_2O using one gas hood at each zone/stage;<br>o One sampling point at each stage of the BNR;<br>o Each sampling point monitored for over 1 day.	[1,2]
A two-stage plug-flow BNR plant with wastewater and returned activated sludge (RAS) both fed to the anoxic zone	➢ 0.036%<br>• A high degree of diurnal and spatial variability in N_2O emission;<br>• N_2O emissions were negatively correlated to dissolved oxygen concentration;	o Eight-week continuous, online monitoring of gaseous N_2O using one gas hood in both the aerobic zone and the anoxic zone;<br>o The gas collection hood was placed for a period of around 1 week at each of the seven sites in the aerobic zone.	[3]
A BNR plant incorporating a plug flow reactor with an MLE configuration, followed by two parallel carousel reactors	➢ 2.8%<br>The nitrous oxide emission exhibited a seasonal dynamic	o The plant was fully covered except for the second clarifier;<br>o The N_2O concentration in the off-gas from the whole plant except for the second clarifier was continuously monitored online over three months.	[4]
An SBR BNR plant	➢ 6.8%<br>• Cycles with long aerated phases showed the highest N_2O emissions;<br>• Cycles with intermittent aeration (an aerated phase up to 20 to 30 min followed by a short anoxic phase) effectively reduce N_2O emissions;	o Continuous online-monitoring over 33 days at one sampling location.	[5]

A separate stage plug–flow BNR	➢ 0.116%<br>• N_2O emission dynamics were highly variable and mainly related to the instability of the nitrification process occurring in the bioreactor;<br>• Transient anoxic periods in the aerated zones resulted in N_2O peak emissions when aeration restarted.	o Continuous online-monitoring using one gas hood in both the aerobic zone and the anoxic zone;<br>o The gas collection hood was placed for a period of around 2–3 days at each of the six sites in the aerobic zone.	6
A pilot WWTP including a few continuous stirred-tank reactors (CSTR), operated as 1) an two-stage MLE system and 2) a three-stage process (pre-denitrification, first oxidation, second oxidation)	➢ 0 - 0.3%<br>• A high degree of diurnal variability in N_2O emission, correlated with diurnal total nitrogen loading;<br>• Higher DO concentration in aerobic tank lead to lower N_2O emission and better resistance to stress conditions favouring higher N_2O emission;<br>• Low sludge ages (10 to12 days) resulted in higher N_2O emissions;<br>• Higher recycle rates (sludge recycle and internal recirculation) contribute to the reduction of N_2O emission.	o Continuous, online monitoring of gaseous N_2O using one gas hood over several months;<br>o One sampling point at each stage of the BNR.	7
Studies with emission factor estimated based on grab sampling			
Activated sludge plant-primary and secondary treatment (aeration only)	➢ 0.035%-0.05%<br>• The most significant emissions occurred in the aerobic zone;<br>• Dissolved N_2O produced by denitrification was stripped during mechanical aeration.	Weekly grab samples for 15 weeks	8
A BNR plant	➢ 0.001%<br>N_2O emissions increased with nitrite and nitrate concentrations	Grab samples in alternate weeks for 1 year	9
Anoxic-aerobic activated sludge plant	➢ 0.001%-0.04%<br>N_2O emission was dependent on the COD:N ratio	Grab samples	10
Intermittently aerated activated sludge plant	➢ 0.01-0.08%<br>N_2O emission decreased with shorter aeration periods	Gas phase N_2O samples collected using air bags during 4 aeration cycles (2 hours)	11

Two intermittently aerated activated sludge plants	➢ 0.47% and 0.01%		12
Seven BNR plants with various configurations	➢ 0.6-25%<br>Large variations in N_2O emissions between plants	Grab samples	13
An anoxic/anaerobic/oxic BNR plant	➢ 0.10%-0.13%<br>The most significant factors influencing N_2O emissions were dissolved oxygen concentration and nitrite concentration in the oxic tanks	Gas phase N_2O samples collected using air bags in oxic tanks, final clarifier tanks, anoxic tanks, sludge concentration tanks and anaerobic tanks	14
Three BNR plants including a pre-anaerobic carrousel oxidation ditch, a pre-anoxic anaerobic-anoxic-oxic process and a reverse anaerobic-anoxic-oxic process	➢ 0.114%-0.140%<br>The nitrite concentration were found to be the dominant influencing factors affecting N_2O production	Grab samples collected with air bags at influent pump station, grit chambers; primary settling tanks, pre-anoxic tanks, anaerobic tanks, anoxic tanks, oxic tanks, final clarifier tanks, et al.	15
an anoxic/anaerobic/oxic BNR plant and a SBR	➢ 6.52% for the SBR plant; 1.9% for the A_2O plant<br>The low DO concentration during nitrification was the major factor influencing N_2O production.	Grab samples-Collecting gas phase N_2O samples using air bags at grit tank, primary clarifier, A_2O-anoxic zone, A_2O-anaerobic zone, A_2O-oxic zone and Final clarifier	16
Oxidation ditch	➢ 0.52%<br>Majority of the N_2O emission was found to occur in the surface aerator zone, which would be missed if the gas hood method was applied alone to quantify N_2O emission.	N_2O emissions was determined based on N_2O transfer coefficient (kLa) induced by surface aerators based on oxygen balance for the entire oxidation ditch.	17

References:

1 Ahn, J. H. *et al.* N_2O emissions from activated sludge processes, 2008-2009: results of a national monitoring survey in the united states. *Environ Sci Technol* **44**, 4505-4511, (2010).

2 Ahn, J. H. *et al.* Spatial and temporal variability in atmospheric nitrous oxide generation and emission from full-scale biological nitrogen removal and non-BNR processes. *Water Environ Res* **82**, 2362-2372, (2010).

3 Aboobakar, A. *et al.* Nitrous oxide emissions and dissolved oxygen profiling in a full-scale nitrifying activated sludge treatment plant. *Water Res* **47**, 524-534, (2013).

4 Daelman, M. R. J., Van Voorthuizen, E. M., Van Dongen, L. G. J. M., Volcke, E. I. P. & Van Loosdrecht, M. C. M. Methane and nitrous oxide emissions from municipal wastewater treatment - Results from a long-term study. *Water Sci Technol* **67**, 2350-2355, (2013).

5 Rodriguez-Caballero, A., Aymerich, I., Marques, R., Poch, M. & Pijuan, M. Minimizing N2O emissions and carbon footprint on a full-scale activated sludge sequencing batch reactor. *Water Res* **71**, 1-10, (2015).

6 Rodriguez-Caballero, A., Aymerich, I., Poch, M. & Pijuan, M. Evaluation of process conditions triggering emissions of green-house gases from a biological wastewater treatment system. *Sci Total Environ* **19**, 384-391, (2014).

7 Lotito, A. M., Wunderlin, P., Joss, A., Kipf, M. & Siegrist, H. Nitrous oxide emissions from the oxidation tank of a pilot activated sludge plant. *Water Res* **46**, 3563-3573, (2012).

8 Czepiel, P., Crill, P. & Harriss, R. Nitrous Oxide Emissions from Municipal Wastewater Treatment. *Environ Sci Technol* **29**, 2352-2356, (1995).

9 Sümer, E., Weiske, A., Benckiser, G. & Ottow, J. C. G. Influence of environmental conditions on the amount of N2O released from activated sludge in a domestic waste water treatment plant. *Cellular and Molecular Life Sciences* **51**, 419-422, (1995).

10 Benckiser, G. *et al.* N2O emissions from different cropping systems and from aerated, nitrifying and denitrifying tanks of a municipal waste water treatment plant. *Biology and Fertility of Soils* **23**, 257-265, (1996).

11 Kimochi, Y., Inamori, Y., Mizuochi, M., Xu, K.-Q. & Matsumura, M. Nitrogen removal and N2O emission in a full-scale domestic wastewater treatment plant with intermittent aeration. *J Ferment Bioeng* **86**, 202-206, (1998).

12 Peu, P., Beline, F., Picard, S. & Heduit, A. in *IWA World Water Congress.* (International Water Association).

13 Foley, J., De Haas, D., Yuan, Z. & Lant, P. Nitrous oxide generation in full-scale biological nutrient removal wastewater treatment plants. *Water Res* **44**, 831-844, (2010).

14 Wang, J. *et al.* Nitrous oxide emissions from a typical northern Chinese municipal wastewater treatment plant. *Desalination and Water Treatment* **32**, 145-152, (2011).

15 Ren, Y. G. *et al.* Nitrous oxide and methane emissions from different treatment processes in full-scale municipal wastewater treatment plants. *Environmental Technology (United Kingdom)* **34**, 2917-2927, (2013).

16 Sun, S. *et al.* N 2 O emission from full-scale urban wastewater treatment plants: a comparison between A 2 O and SBR. *Water Sci Technol* **67**, 1887-1893, (2013).

17 Ye, L., Ni, B.-J., Law, Y., Byers, C. & Yuan, Z. A novel methodology to quantify nitrous oxide emissions from full-scale wastewater treatment systems with surface aerators. *Water Res* **48**, 257-268, (2014).
